# Excess Uric Acid Induces Gouty Nephropathy Through Crystal Formation: A Review of Recent Insights

**DOI:** 10.3389/fendo.2022.911968

**Published:** 2022-07-14

**Authors:** Yongsheng Mei, Bingzi Dong, Zhuang Geng, Lili Xu

**Affiliations:** Department of Endocrinology and Metabolism, The Affiliated Hospital of Qingdao University, Qingdao, China

**Keywords:** uric acid, hyperuricemia, gout, gouty nephropathy, treatment

## Abstract

Uric acid (UA) is the final product of purine metabolism in the human body, and impaired purine metabolism can increase the uric acid in serum, finally resulting in hyperuricemia (HUA). Current evidences suggest that urates might have antioxidant properties under certain circumstances, but most evidences suggest that urates promote inflammation. Hyperuricemia leads to the formation of urate crystals, which might be recognized as a red flag by the immune system. Such a response stimulates macrophage activation, leads to the activation of NOD-like receptor protein 3 (NLRP3) inflammasome vesicles, and ultimately the production and liberation of interleukin-1b (IL-1b) and interleukin-18 (IL-18), which can mediate inflammation, apoptosis and necroinflammation and cause an inflammatory cascade response. The kidney is one of the most commonly affected organs in HUA, which promotes the development of chronic kidney disease (CKD) by damaging endothelial cells, activating the renin-angiotensin system (RAS), and promoting inflammatory responses. Pharmacological interventions and lifestyle modifications are the primary means for controlling gout and lowering UA. The febuxostat is safe for CKD patients in the UA lowering therapy. Although dialysis can reduce UA levels, the application of drug is also necessary for dialysis patients. This article reviews the synthesis and metabolism of UA, etiology of HUA, the relationship between HUA and kidney disease, the treatment of gout and gouty nephropathy (GN).

## Introduction

Uric acid is the final metabolic product of purines in humans. As the final product of exogenous purines from food and endogenous purines from damaged and dead cells, uric acid is synthesized mainly in the liver, intestine and vascular endothelium (1). The kidney plays a leading position in the excretion of uric acid, with about 70% of the uric acid produced daily being excreted by the kidneys; as the remaining 30% is excreted from the intestines (1). After filtration by the glomerulus, uric acid is absorbed, secreted and reabsorbed by the proximal tubule, and the unabsorbed portion is excreted in the urine. In the proximal tubule, reabsorbed urate is secreted into the tubular lumen, about 10% of the filtered urate is excreted in the urine, and the rest 90% is reabsorbed (2).

Under physiological conditions, the synthesis and excretion of uric acid in our body is in balance. Hyperuricemia results when this balance is disturbed. Typically, levels of serum uric acid >6.8 mg/dl are considered to be hyperuricemia (3). The overall prevalence of hyperuricemia in China is 13.3%, and the prevalence of gout is 1.1%, Hyperuricemia is more common in men than in women; UA levels in women of reproductive age are lower than their male counterparts due to the inhibition of renal urate reabsorption with an increased renal urate clearance by estrogenic compounds (4). Elevated plasma uric acid is caused by either overproduction or decreased excretion. Overproduction is usually idiopathic and may also occur as a result of increased purine release due to massive tissue destruction, such as tumor lysis syndrome, crush injuries or intractable epilepsy. Overproduction may also be caused by genetic enzyme defects and reduced excretion may be idiopathic and related to drugs (e.g., thiazide diuretics, cyclosporine A). The dietary factor plays an important part in the development of hyperuricemia (5), as purine eventually degrades into uric acid; excessive consumption of alcohol and purine-rich foods (such as red meat, seafood, some vegetables, and animal proteins) is associated with the development of hyperuricemia. Dairy product intake is negatively associated with serum urate concentration (3). The development of hyperuricemia has been shown to be associated with multiple genetic factors and the uric acid transporter protein genes SLC2A9 (encoding GLUT9), SLC22A12 (encoding URAT1), SLC17A1 (encoding NPT1) and ABCG2 were most strongly correlated with changes in serum uric acid levels (6–9). Therefore, by detecting pathogenicity associated with urate crystals, gene assay can screen for high risk of gout in hyperuricemia patients.

When the level of serum uric acid exceeds the solubility threshold, uric acid precipitates into crystalline urate crystals, which manifest as acute episodes of painful arthritis, forming gout (10). Studies have found that a variety of factors influence the information of urate crystals, such as temperature, sodium ion concentration, pH, mechanical stress, cartilage composition, uric acid binding antibodies, cartilage and synovial fluid composition (11). Although some patients do not relapse after the first episode, the majority progress naturally, showing chronic inflammation, frequent attacks, gout stone formation and joint destruction (12). Only about 2-6% of patients with hyperuricemia progress to gout (3), but the mechanism by which most patients with hyperuricemia do not develop gout is not yet understood (13). A multi-stage genome-wide association study (GWAS) identified three loci, 17q23.2 (rs11653176, BCAS3), 9p24.2 (rs12236871, RFX3) and 11p15.5 (rs179785, KCNQ1), which contain inflammatory candidate genes and are likely to be associated with the development from hyperuricemia to gout (14). Another GWAS study showed that three loci (CNTN5, MIR302F and ZNF724) were related to the mechanism of gout development (15), which is different from the gout risk loci that raise serum uric acid levels we know now. In this review, we focus on the role that uric acid and gout play in kidney disease and the problems currently encountered in the treatment of gouty nephropathy.

## Uric Acid - Anti-Inflammatory, or Pro-Inflammatory?

It has been shown that urate has properties to scan for free radicals and has strong antioxidant capacity in human body (16). Uric acid positively affects neurological function by inhibiting the accumulation of oxygen free radicals, stabilizing calcium homeostasis, maintaining mitochondrial function and protecting neurons from glutamate-related toxicity. Against the data supporting the anti-inflammatory effects of urate, urate has been found to be a pro-oxidant, forming free radicals in the reaction with other oxidants that appear to target mainly lipids (e.g., low-density lipoprotein (LDL)) (16). Uric acid stimulates nicotinamide adenine dinucleotide phosphate (NADPH) oxidase-dependent reactive oxygen species (ROS), leading to mitogen-activated protein kinase (MAPK) kinase p38 and extracellular regulated protein kinases (ERK) 1/2 activation, decreased nitric oxide bioavailability, and increased protein nitrosylation and lipid oxidation (17). The limited prospective data do not clearly support the potential antioxidant and organ-protective effects of urate. On the contrary, when the pro-inflammatory effects of urate exceed the anti-inflammatory effect, especially as its dissolution exceeds the limit (>6.8 mg/dL), gout occurs. The results of several observational studies have shown that hyperuricemia is associated with hypertension (18) and heart failure (19).

## Gout and Kidney Disease

**Figure 1:**
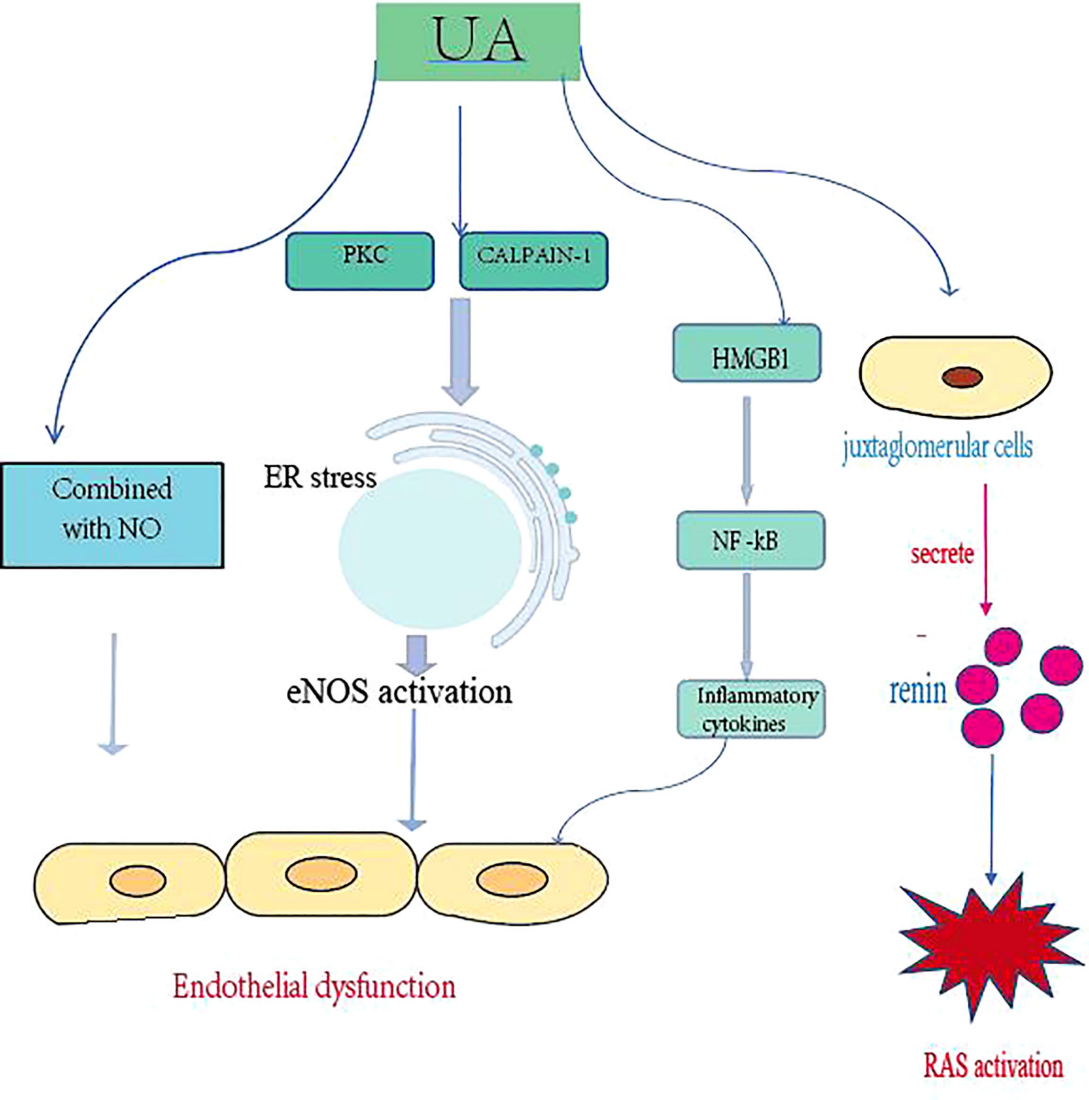
Uric acid promotes the development of CKD by damaging endothelial cells, activating the RAS, and promoting inflammatory responses. PKC, protein kinase C; ER, endoplasmic reticulum; NOS, endothelial nitric oxide synthase; HMGB1, high mobility group box chromosomal protein 1; NF-kB, nuclear factor kB; RAS, renin-angiotensin system.

Gouty nephropathy, also known as uric acid nephropathy, is a series of kidney disorders caused by an increase in uric acid in the human serum, which accumulates in the renal tubules and interstitium over a long period of time. Renal damage is a common comorbidity of gout and as kidney function declines, uric acid excretion through the urine is reduced, leading to hyperuricemia. Persistent hyperuricemia leads to the formation of urate crystals in joints and tissues (20). A recent meta-analysis estimated that 24% (95% confidence interval 15 - 28) of gout patients exhibited chronic kidney disease beyond stage 3 (21). Hyperuricemia also often occurs in advanced CKD, with a prevalence of 64% in patients with stage 3 CKD and 50% in patients with stage 4 or 5 CKD (22). In a representative national study in the USA, 19.9% of gout adults had CKD ≥ stage 3, in contrast to 5.2% in adults without gout (23). Uric acid induces hypertension by affecting endothelial function and impaired nitric oxide production, and hypertension may be the initial trigger for subclinical renal damage. UA significantly increased the production of reactive oxygen species and angiotensin II, inducing senescence and apoptosis of endothelial cells at concentrations above 6 and 9 mg/dL, respectively. Hyperuricemia may also lead to microvascular injury by stimulating the renin-angiotensin system (RAS) (24), inhibiting endothelial-type nitric oxide and vascular smooth muscle proliferative effects (**Figure 1**). Hyperuricemia increases renin expression in glomerular cells and (pro)renin receptor expression in endothelial cells, while decreasing nitric oxide synthase-1 expression in the macula. The formation of urate crystals in hyperuricemia causes gout attacks largely through activation of monocytes and macrophages, generates NLRP3 inflammatory vesicle-mediated interleukin (IL)-1β release, and leads to many other local and systemic high-level pro-inflammatory responses and joint neutrophil in-flow and activation (25). Consistent with the findings of urate crystals, Braga et al. showed that soluble uric acid salts also activate NLRP3 inflammatory vesicles and induce IL-1β production. This proinflammatory effect of uric acid on tubular cells works through High mobility group box chromosomal protein 1 (HMGB1) release and nuclear factor kappa-B (NF-κB) signaling activation (26). In this context, the hypo-inflammatory phenotype in CKD has been confirmed by several studies, which are associated with increased concentrations of serum C-reactive protein, multiple pro-inflammatory cytokines, prostaglandins and leukotrienes, and dysbiosis of the intestinal flora (27). In addition, cumulative data suggest that treatment to reduce UA may slow the progression of these diseases.

## Pathogenesis of Gouty Nephropathy

The mechanism of GN is mainly related to hyperuricemia and the deposition of monosodium urate crystals in the body. Survival conditions comprise high purine intake, excessive obesity and high dietary fructose concentration drinks, combined with the abuse of some drugs that affect the metabolic process of acid, such as thiazide diuretics, salicylates, and other metabolic substances such as lactic acid, ketone bodies and angiotensin. Excess uric acid deposited in the capillaries greatly increases the burden on the kidneys, leaving them in a state of long-term compensatory work, which eventually leads to a decrease in the filtration function of the kidneys and the deposition of urate crystals in the kidneys, causing lesions. Monosodium urate crystals precipitate in the renal tubules (usually the collecting ducts), causing acute gouty nephropathy. Uric acid stones may develop in 15-20% of patients with acute gouty nephropathy (28). Chronic gouty nephropathy is associated with urate crystal deposits and is mainly seen in patients suffer from gout, hypertension. The characteristic histological features of uric acid nephropathy are the presence of urate deposits in the interstitium and tubules, which can be seen as birefringent, needle-like urate crystals. Microcalcifications in the collecting ducts can cause dilatation of the collecting ducts and predispose to secondary bacterial infections. It is also associated with endothelial cell damage, activation of the renin-angiotensin system (RAS), induction of inflammatory responses by monosodium urate crystals, and activation of the cyclooxygenase (COX-2) system. A cross-sectional study of 502 patients found that the renal medulla of patients with severe gout was diffusely hyperechoic (29). This finding supports the idea that the renal medulla of patients with long-term untreated gout is echogenic, which may be associated with the development of gout stones within the renal medulla. This nephropathy is not the only mechanism of hyperuricemia and gouty kidney damage; other factors, such as vascular involvement and non-steroidal anti-inflammatory drugs occur in 15-20% of patients with acute gouty nephropathy. Acute gouty nephropathy shows clusters of urate crystals in the aggregated tubular lumen with acute tubular damage. Needle-like birefringent crystals of sodium urate are seen on alcohol-fixed or frozen sections. These crystals dissolve during paraffin-embedded tissue processing and form needle-like fissures. Chronic gouty nephropathy presents as intra-tubular and/or interstitial microliths consisting of a central needle-like cleft surrounded by cellular reactions including epithelioid macrophages, lymphocytes and eosinophils, accompanied by tubular interstitial fibrosis. Glomerular changes include thylakoid stromal hyperplasia and double contouring of the glomerular basement membranes (28).

## Treatment of Gout and Gout Nephropathy

The main goal of gout treatment is to remove all urate crystals by lowering uric acid levels below 6 mg/dL. The process of deposition of urate crystals is reversible; crystals continue to form in gout patients and persist in patients with hyperuricemia, but dissolve when serum uric acid is lowered below the saturation point and the associated inflammation subsides with the disappearance of urate crystals (30). Importantly, lower uric acid levels lead to an accelerated rate of crystal dissolution: serum uric acid values below 4 mg/dL reduce the diameter of gout stones at a rate twice as fast as serum uric acid values above 5 mg/dL (31). Early treatment can lead to easier improvement. The 2020 American College of Rheumatology (ACR) guidelines recommend colchicine, non-steroidal anti-inflammatory drugs (NSAIDs), and non-gut/oral glucocorticoids as first-line treatment options for gout (32). However, treatment options for gout attacks with minimal or no residual renal function are limited, with the potential risk of further renal impairment. Corticosteroids have been recognized as the safest option for most patients with gout attacks and CKD (25). Short-term use of glucocorticoids is considered an acceptable risk and long-term use of glucocorticoids can lead to an increased risk of associated adverse events, particularly infections (33). The use of NSAIDs is a very common treatment for gout attacks but is not indicated for patients with renal injury (RI) and many comorbidities in older adults. In the AGREE clinical trial, low-dose colchicine was comparable to high-dose colchicine for the treatment of gout attacks with minimal side effects; therefore, low-dose colchicine has been recommended for the prevention and treatment of gout attacks (34). However, the use of colchicine for the treatment of gout attacks in RI patients has been banned (35). There are several randomized controlled trials (RCT) on colchicine to treat gout attacks, none of which have results stratified by renal function (36). In case reports and case series, we see different results on the effects of gout attack treatment on renal function. For example, 12 studies reported worsening renal function with colchicine, while another seven studies reported stable renal function with colchicine (37). All NSAIDs are widely regarded as contraindications in advanced CKD, and those patients with renal failure are usually given intraarticular or systemic steroids.

For gout sufferers, dietary control is advocated. Reduce the consumption of high purine foods such as animal offal, red meat, sugar, seafood, soda and alcoholic beverages. Eat plenty of vegetables, vitamin C, skimmed milk, low-fat yoghurt, soy products, drink plenty of water (keep daily urine output above 2000ml), and avoid full meals. Alkalinize urine to pH 6.2-6.5 (excessive alkalinization tends to form calcium phosphate or calcium carbonate stones). Lifestyle modifications: exercise properly, lower body mass, drink green tea, maintain proper hunger (1 hour of hunger rests organs and increases longevity genes), promote early dinner, and in addition, educate patients properly to improve compliance and treatment outcomes.

## The use of Uric Acid-Lowering Drugs in Patients With Gout

There are two main types of drugs that are commonly used clinically to lower blood uric acid (ULT): XO inhibitors that inhibit uric acid synthesis, such as allopurinol and febuxostat, and drugs that improve the excretion of uric acid (benzbromarone, etc.). Compared with allopurinol, previous studies have shown febuxostat to be more effective and safer. Recent studies have shown that ULT has no effect on the occurrence of the primary endpoint events (cardiovascular death, non-fatal myocardial infarction, non-fatal stroke, or unstable angina combined with emergency revascularization). XO inhibitors have been reported to be beneficial in CKD. A meta-analysis showed that XO inhibitors reduced the risk of end stage renal disease significantly and also improved estimated glomerular filtration rate (eGFR) from data from randomized controlled trials with long-term follow-up (>3 months) (38). Further studies are needed to elucidate the effect of non-purine XO inhibitors on the development and progression of CKD. However, current meta-analyses do not demonstrate a nephroprotective effect of ULT. CKD is shown to lead to an increased risk of cardiovascular disease (CVD) in the general population (39). The use of ULT in patients with advanced CKD varies considerably among rheumatologists, nephrologists and general practitioners (40), and the proper use of ULT in patients with gout and CKD is controversial. The ACR, the European Rheumatism Association and the British Society for Rheumatology have published the updated guidelines with difference in some important areas, such as allopurinol dosing in patients with CKD (32, 41, 42). Allopurinol is metabolized in the liver to active allopurinol and excreted by the kidneys. When the kidneys are not functioning properly, allopurinol tends to accumulate in the body, increasing the risk of drug toxicity. There are two main reasons for avoiding the use of ULT in patients with CKD: lack of efficacy and increased risk of adverse events. In general, patients with eGFR <30 ml/min/1.73 m^2^ are reluctant to use allopurinol because of the potentially fatal allopurinol hypersensitivity syndrome (AHS) and the poor prognosis for patients with impaired renal function who develop AHS (43). Febuxostat is a non-purine selective xanthine oxidase inhibitor, metabolized mainly in the liver by glucosylation, and its use in CKD has become more widely accepted. In one of the largest studies of febuxostat for CKD, which included 96 patients with glomerular filtration rates (eGFR) in the range of 15 - 50 ml/min/1.73 m^2^, febuxostat 60 - 80 mg/day was associated with a reduction in serum urate concentrations (compared with placebo) but no reduction in renal function (44), which may suggest that febuxostat is effective in reducing serum uric acid and is well tolerated in patients with moderate to severe renal insufficiency in gout. It is not clear whether the rate of gout attacks in patients with CKD at the time of initiation of ULT is the same as in those without ULT and whether prophylaxis is always required (45).

Regarding the cardiovascular safety of febuxostat versus allopurinol in patients with gout and CVD, the use of febuxostat in CVD patients has been controversial. CARES conducted a large randomized controlled trial (RCT) in the United States in patients with gout and pre-existing cardiovascular disease. CARES randomly assigned 6190 patients with gout and cardio-vascular disease to receive febuxostat or allopurinol and patients were stratified according to kidney function. The CARES study found that all-cause and cardiovascular mortality were higher in the febuxostat group than in the allopurinol group (hazard ratio for death from any cause, 1.22 [95% CI, 1.01 to 1.47]; hazard ratio for cardiovascular death, 1.34 [95% CI, 1.03 to 1.73]) (46). FAST is another large randomized controlled trial conducted in European countries to compare the cardiovascular safety of febuxostat versus allopurinol in patients with gout. The primary endpoint of febuxostat was not lower than that of allopurinol. In contrast to the CARES trial, FAST found that febuxostat treatment was not associated with increased cardiovascular death or all-cause mortality, and mortality in the febuxostat group was lower than in the allopurinol group. In the febuxostat group, 222 (7.2%) of 3063 patients died and 1720 (57.3%) of 3001 in the safety analysis set had at least one serious adverse event (with 23 events in 19 [0.6%] patients related to treatment). In the allopurinol group, 263 (8.6%) of 3065 patients died and 1812 (59.4%) of 3050 had one or more serious adverse events (with five events in five [0.2%] patients related to treatment) (30). Although the two studies were of similar size, there were still several differences. Only 33% of patients in FAST had cardiovascular disease at baseline while all patients in CARES had established cardiovascular disease. FAST had more complete follow-up than CARES because they used telephone or personal contact and national hospitalization and death records. In consideration of these findings, we should reconsider the use of febuxostat in patients with cardiovascular disease.

## Dialysis and Uric Acid

Dialysis provides appropriate clearance of serum uric acid. Relevant studies have shown that the average uric acid in hemodialysis patients is less than 5 mg/dL and the average serum uric acid after dialysis is less than 1 mg/dL, suggesting that the initiation of hemodialysis leads to clearance of tophus (43). It has been shown that serum urate reaches target concentrations less frequently in hemodialysis patients than in peritoneal dialysis patients, possibly because dialysis removes urate intermittently rather than continuously (47). It is also shown that ULT should be considered as dialysis alone is not enough to achieve ideal serum urate levels for patients (48). Allopurinol, the active metabolite of allopurinol, has been shown to be effective in reducing serum uric acid in hemodialysis patients.

## Conclusion

Much of the current knowledge of the biological role of uric acid comes from experimental studies that have revealed that uric acid is associated with immune system activation and inflammation hyperuricemia may play a key role in the development and progression of CKD. Available evidence suggests that uric acid reduction therapy may slow the progression of CKD, although the molecular mechanism of uric acid-induced gout nephropathy remains to be further understood. Gout patients should be screened for renal function and clinicians should be aware of the link between gout and impaired renal function. The use of ULT drugs remain controversial. Long-term dietary control, lifestyle modification and patient education are the cornerstones of treatment.

## Author Contributions

YM contributed to the conception and the writing of the article. BD performed the framework. ZG gave the constructive discussions to the article. LX revised important intellectual content critically for important intellectual content. All authors contributed to the article and approved the submitted version.

## Conflict of Interest

The authors declare that the research was conducted in the absence of any commercial or financial relationships that could be construed as a potential conflict of interest.

## Publisher’s Note

All claims expressed in this article are solely those of the authors and do not necessarily represent those of their affiliated organizations, or those of the publisher, the editors and the reviewers. Any product that may be evaluated in this article, or claim that may be made by its manufacturer, is not guaranteed or endorsed by the publisher.

## References

[1] Yanai H Adachi H Hakoshima M Katsuyama H. Molecular Biological and Clinical Understanding of the Pathophysiology and Treatments of Hyperuricemia and Its Association With Metabolic Syndrome, Cardiovascular Diseases and Chronic Kidney Disease. Int J Mol Sci (2021) 22(17):9221. doi: 10.3390/ijms22179221 34502127 PMC8431537

[2] Maiuolo J Oppedisano F Gratteri S Muscoli C Mollace V. Regulation of Uric Acid Metabolism and Excretion. Int J Cardiol (2016) 213:8–14. doi: 10.1016/j.ijcard.2015.08.109 26316329

[3] Keenan RT. The Biology of Urate. Semin Arthritis Rheumatol (2020) 50(3S):S2–S10. doi: 10.1016/j.semarthrit.2020.04.007 32620198

[4] Liu R Han C Wu D Xia X Gu J Guan H. Prevalence of Hyperuricemia and Gout in Mainland China From 2000 to 2014: A Systematic Review and Meta-Analysis. BioMed Res Int (2015) 2015:762820. doi: 10.1155/2015/762820 26640795 PMC4657091

[5] MacFarlane LA Kim SC. Gout: A Review of Nonmodifiable and Modifiable Risk Factors. Rheum Dis Clin North Am (2014) 40(4):581–604. doi: 10.1016/j.rdc.2014.07.002 25437279 PMC4251556

[6] Major TJ Dalbeth N Stahl EA Merriman TR. An Update on the Genetics of Hyperuricaemia and Gout. Nat Rev Rheumatol (2018) 14(6):341–53. doi: 10.1038/s41584-018-0004-x 29740155

[7] Okada Y Sim X Go MJ Wu JY Gu D Takeuchi F. Meta-Analysis Identifies Multiple Loci Associated With Kidney Function-Related Traits in East Asian Populations. Nat Genet (2012) 44(8):904–9. doi: 10.1038/ng.2352 PMC4737645 22797727

[8] Dehghan A van Hoek M Sijbrands EJ Hofman A Witteman JC. High Serum Uric Acid as a Novel Risk Factor for Type 2 Diabetes. Diabetes Care (2008) 31(2):361–2. doi: 10.2337/dc07-1276 17977935

[9] Anzai N Jutabha P Amonpatumrat-Takahashi S Sakurai H. Recent Advances in Renal Urate Transport: Characterization of Candidate Transporters Indicated by Genome-Wide Association Studies. Clin Exp Nephrol (2012) 16(1):89–95. doi: 10.1007/s10157-011-0532-z 22038265

[10] Hansildaar R Vedder D Baniaamam M Tausche A-K Gerritsen M Nurmohamed MT. Cardiovascular Risk in Inflammatory Arthritis: Rheumatoid Arthritis and Gout. Lancet Rheumatol (2021) 3(1):e58–70. doi: 10.1016/S2665-9913(20)30221-6 PMC7462628 32904897

[11] Chhana A Pool B Callon KE Tay ML Musson D Naot D. Monosodium Urate Crystals Reduce Osteocyte Viability and Indirectly Promote a Shift in Osteocyte Function Towards a Proinflammatory and Proresorptive State. Arthritis Res Ther (2018) 20(1):208. doi: 10.1186/s13075-018-1704-y 30201038 PMC6131786

[12] Ragab G Elshahaly M Bardin T. Gout: An Old Disease in New Perspective - A Review. J Adv Res (2017) 8(5):495–511. doi: 10.1016/j.jare.2017.04.008 28748116 PMC5512152

[13] Cabau G Crisan TO Kluck V Popp RA Joosten LAB. Urate-Induced Immune Programming: Consequences for Gouty Arthritis and Hyperuricemia. Immunol Rev (2020) 294(1):92–105. doi: 10.1111/imr.12833 31853991 PMC7065123

[14] Li C Li Z Liu S Wang C Han L Cui L. Genome-Wide Association Analysis Identifies Three New Risk Loci for Gout Arthritis in Han Chinese. Nat Commun (2015) 6:7041. doi: 10.1038/ncomms8041 25967671 PMC4479022

[15] Kawamura Y Nakaoka H Nakayama A Okada Y Yamamoto K Higashino T. Genome-Wide Association Study Revealed Novel Loci Which Aggravate Asymptomatic Hyperuricaemia Into Gout. Ann Rheum Dis (2019) 78(10):1430–7. doi: 10.1136/annrheumdis-2019-215521 PMC6788923 31289104

[16] Sautin YY Johnson RJ. Uric Acid: The Oxidant-Antioxidant Paradox. Nucleosides Nucleotides Nucleic Acids (2008) 27(6):608–19. doi: 10.1080/15257770802138558 PMC2895915 18600514

[17] Sautin YY Nakagawa T Zharikov S Johnson RJ. Adverse Effects of the Classic Antioxidant Uric Acid in Adipocytes: NADPH Oxidase-Mediated Oxidative/Nitrosative Stress. Am J Physiol Cell Physiol (2007) 293(2):C584–96. doi: 10.1152/ajpcell.00600.2006 17428837

[18] Wang J Qin T Chen J Li Y Wang L Huang H. Hyperuricemia and Risk of Incident Hypertension: A Systematic Review and Meta-Analysis of Observational Studies. PLoS One (2014) 9(12):e114259. doi: 10.1371/journal.pone.0114259 25437867 PMC4250178

[19] Duan X Ling F. Is Uric Acid Itself a Player or a Bystander in the Pathophysiology of Chronic Heart Failure? Med Hypotheses (2008) 70(3):578–81. doi: 10.1016/j.mehy.2007.06.018 17689199

[20] Pascual E Addadi L Andres M Sivera F. Mechanisms of Crystal Formation in Gout-a Structural Approach. Nat Rev Rheumatol (2015) 11(12):725–30. doi: 10.1038/nrrheum.2015.125 26369610

[21] Roughley MJ Belcher J Mallen CD Roddy E. Gout and Risk of Chronic Kidney Disease and Nephrolithiasis: Meta-Analysis of Observational Studies. Arthritis Res Ther (2015) 17:90. doi: 10.1186/s13075-015-0610-9 25889144 PMC4404569

[22] Krishnan E. Reduced Glomerular Function and Prevalence of Gout: NHANES 2009-10. PLoS One (2012) 7(11):e50046. doi: 10.1371/journal.pone.0050046 23209642 PMC3507834

[23] Zhu Y Pandya BJ Choi HK. Comorbidities of Gout and Hyperuricemia in the US General Population: NHANES 2007-2008. Am J Med (2012) 125(7):679–87.e1. doi: 10.1016/j.amjmed.2011.09.033 22626509

[24] Mallat SG Al Kattar S Tanios BY Jurjus A. Hyperuricemia, Hypertension, and Chronic Kidney Disease: An Emerging Association. Curr Hypertens Rep (2016) 18(10):74. doi: 10.1007/s11906-016-0684-z 27696189

[25] Stamp LK Farquhar H Pisaniello HL Vargas-Santos AB Fisher M Mount DB. Management of Gout in Chronic Kidney Disease: A G-CAN Consensus Statement on the Research Priorities. Nat Rev Rheumatol (2021) 17(10):633–41. doi: 10.1038/s41584-021-00657-4 PMC8458096 34331037

[26] Braga TT Forni MF Correa-Costa M Ramos RN Barbuto JA Branco P. Soluble Uric Acid Activates the NLRP3 Inflammasome. Sci Rep (2017) 7:39884. doi: 10.1038/srep39884 28084303 PMC5233987

[27] Mihai S Codrici E Popescu ID Enciu AM Albulescu L Necula LG. Inflammation-Related Mechanisms in Chronic Kidney Disease Prediction, Progression, and Outcome. J Immunol Res (2018) 2018:2180373. doi: 10.1155/2018/2180373 30271792 PMC6146775

[28] Lusco MA Fogo AB Najafian B Alpers CE. AJKD Atlas of Renal Pathology: Gouty Nephropathy. Am J Kidney Dis (2017) 69(1):e5–6. doi: 10.1053/j.ajkd.2016.11.006 28007197

[29] Bardin T Nguyen QD Tran KM Le NH Do MD Richette P. A Cross-Sectional Study of 502 Patients Found a Diffuse Hyperechoic Kidney Medulla Pattern in Patients With Severe Gout. Kidney Int (2021) 99(1):218–26. doi: 10.1016/j.kint.2020.08.024 32898570

[30] Mackenzie IS Ford I Nuki G Hallas J Hawkey CJ Webster J. Long-Term Cardiovascular Safety of Febuxostat Compared With Allopurinol in Patients With Gout (FAST): A Multicentre, Prospective, Randomised, Open-Label, non-Inferiority Trial. Lancet (2020) 396(10264):1745–57. doi: 10.1016/S0140-6736(20)32234-0 33181081

[31] Perez-Ruiz F Calabozo M Pijoan JI Herrero-Beites AM Ruibal A. Effect of Urate-Lowering Therapy on the Velocity of Size Reduction of Tophi in Chronic Gout. Arthritis Rheumatol (2002) 47(4):356–60. doi: 10.1002/art.10511 12209479

[32] FitzGerald JD Dalbeth N Mikuls T. 2020 American College of Rheumatology Guideline for the Management of Gout. Am Coll Rheumatol (2020) 2020:1–17. doi: 10.1002/acr.24180 32390306

[33] George MD Baker JF Winthrop K Hsu JY Wu Q Chen L. Risk for Serious Infection With Low-Dose Glucocorticoids in Patients With Rheumatoid Arthritis : A Cohort Study. Ann Intern Med (2020) 173(11):870–8. doi: 10.7326/M20-1594 PMC8073808 32956604

[34] Terkeltaub RA Furst DE Bennett K Kook KA Crockett RS Davis MW. High Versus Low Dosing of Oral Colchicine for Early Acute Gout Flare: Twenty-Four-Hour Outcome of the First Multicenter, Randomized, Double-Blind, Placebo-Controlled, Parallel-Group, Dose-Comparison Colchicine Study. Arthritis Rheumatol (2010) 62(4):1060–8. doi: 10.1002/art.27327 20131255

[35] Bardin T Richette P. Impact of Comorbidities on Gout and Hyperuricaemia: An Update on Prevalence and Treatment Options. BMC Med (2017) 15(1):123. doi: 10.1186/s12916-017-0890-9 28669352 PMC5494879

[36] Pisaniello HL Fisher MC Farquhar H Vargas-Santos AB Hill CL Stamp LK. Efficacy and Safety of Gout Flare Prophylaxis and Therapy Use in People With Chronic Kidney Disease: A Gout, Hyperuricemia and Crystal-Associated Disease Network (G-CAN)-Initiated Literature Review. Arthritis Res Ther (2021) 23(1):130. doi: 10.1186/s13075-021-02416-y 33910619 PMC8080370

[37] Farquhar H Vargas-Santos AB Pisaniello HL Fisher M Hill C Gaffo AL. Efficacy and Safety of Urate-Lowering Therapy in People With Kidney Impairment: A GCAN-Initiated Literature Review. Rheumatol Adv Pract (2021) 5(1):rkaa073. doi: 10.1093/rap/rkaa073 33521512 PMC7819867

[38] Pisano A Cernaro V Gembillo G D'Arrigo G Buemi M Bolignano D. Xanthine Oxidase Inhibitors for Improving Renal Function in Chronic Kidney Disease Patients: An Updated Systematic Review and Meta-Analysis. Int J Mol Sci (2017) 18(11):2283. doi: 10.3390/ijms18112283 PMC5713253 29088122

[39] Ali S Dave N Virani SS Navaneethan SD. Primary and Secondary Prevention of Cardiovascular Disease in Patients With Chronic Kidney Disease. Curr Atheroscler Rep (2019) 21(9):32. doi: 10.1007/s11883-019-0794-6 31230129

[40] Stamp LK Taylor W Gaffo A Gout, Crystal Arthritis N. Variability in Urate-Lowering Therapy Prescribing: A Gout, Hyperuricemia and Crystal-Associated Disease Network (G-CAN) Physician Survey. J Rheumatol (2021) 48(1):152–3. doi: 10.3899/jrheum.200347 33004535

[41] Hui M Carr A Cameron S Davenport G Doherty M Forrester H. The British Society for Rheumatology Guideline for the Management of Gout. Rheumatol (Oxford) (2017) 56(7):e1–e20. doi: 10.1093/rheumatology/kex156 28549177

[42] Richette P Doherty M Pascual E Barskova V Becce F Castaneda-Sanabria J. 2016 Updated EULAR Evidence-Based Recommendations for the Management of Gout. Ann Rheum Dis (2017) 76(1):29–42. doi: 10.1136/annrheumdis-2016-209707 27457514

[43] Pascual E Sivera F Andres M. Managing Gout in the Patient With Renal Impairment. Drugs Aging (2018) 35(4):263–73. doi: 10.1007/s40266-018-0517-7 29435850

[44] Saag KG Whelton A Becker MA MacDonald P Hunt B Gunawardhana L. Impact of Febuxostat on Renal Function in Gout Patients With Moderate-To-Severe Renal Impairment. Arthritis Rheumatol (2016) 68(8):2035–43. doi: 10.1002/art.39654 26894653

[45] Yamanaka H Tamaki S Ide Y Kim H Inoue K Sugimoto M. Stepwise Dose Increase of Febuxostat is Comparable With Colchicine Prophylaxis for the Prevention of Gout Flares During the Initial Phase of Urate-Lowering Therapy: Results From FORTUNE-1, a Prospective, Multicentre Randomised Study. Ann Rheum Dis (2018) 77(2):270–6. doi: 10.1136/annrheumdis-2017-211574 PMC5867413 29102957

[46] White WB Saag KG Becker MA Borer JS Gorelick PB Whelton A. Cardiovascular Safety of Febuxostat or Allopurinol in Patients With Gout. N Engl J Med (2018) 378(13):1200–10. doi: 10.1056/NEJMoa1710895 29527974

[47] Yeo E Palmer SC Chapman PT Frampton C Stamp LK. Serum Urate Levels and Therapy in Adults Treated With Long-Term Dialysis: A Retrospective Cross-Sectional Study. Intern Med J (2019) 49(7):838–42. doi: 10.1111/imj.14163 30426652

[48] Wright DF Doogue MP Barclay ML Chapman PT Cross NB Irvine JH. A Population Pharmacokinetic Model to Predict Oxypurinol Exposure in Patients on Haemodialysis. Eur J Clin Pharmacol (2017) 73(1):71–8. doi: 10.1007/s00228-016-2133-y 27683090

